# Indirect epigenetic testing identifies a diagnostic signature of cardiomyocyte DNA methylation in heart failure

**DOI:** 10.1007/s00395-022-00954-3

**Published:** 2023-03-20

**Authors:** Christian U. Oeing, Mark E. Pepin, Kerstin B. Saul, Ayça Seyhan Agircan, Yassen Assenov, Tobias S. Merkel, Farbod Sedaghat-Hamedani, Tanja Weis, Benjamin Meder, Kaomei Guan, Christoph Plass, Dieter Weichenhan, Dominik Siede, Johannes Backs

**Affiliations:** 1 Institute of Experimental Cardiology, University Hospital Heidelberg, University of Heidelberg and DZHK (German Centre for Cardiovascular Research), Partner Site Heidelberg/Mannheim, Im Neuenheimer Feld 669, 69120 Heidelberg, Germany; 2 Department of Internal Medicine and Cardiology, Charité University Medicine, DZHK (German Center for Cardiovascular Research), Partner site Berlin, Campus Virchow-Klinikum, Berlin, Germany; 3 Cancer Epigenomics, German Cancer Research Centre (DKFZ), Heidelberg, Germany; 4 Department of Cardiology, University of Heidelberg, DZHK (German Centre for Cardiovascular Research), Partner Site Heidelberg/Mannheim, Heidelberg, Germany; 5 Institute of Pharmacology and Toxicology, Technische Universität Medical Centre Dresden, Dresden, Germany

**Keywords:** Precision medicine, Epigenetics, Heart failure, DNA methylation, Pilot study

## Abstract

Precision-based molecular phenotyping of heart failure must overcome limited access to cardiac tissue. Although epigenetic alterations have been found to underlie pathological cardiac gene dysregulation, the clinical utility of myocardial epigenomics remains narrow owing to limited clinical access to tissue. Therefore, the current study determined whether patient plasma confers indirect phenotypic, transcriptional, and/or epigenetic alterations to ex vivo cardiomyocytes to mirror the failing human myocardium. Neonatal rat ventricular myocytes (NRVMs) and single-origin human induced pluripotent stem cell-derived cardiomyocytes (*hiPSC-CMs*) and were treated with blood plasma samples from patients with dilated cardiomyopathy (DCM) and donor subjects lacking history of cardiovascular disease. Following plasma treatments, NRVMs and *hiPSC-CMs* underwent significant hypertrophy relative to non-failing controls, as determined via automated high-content screening. Array-based DNA methylation analysis of plasma-treated *hiPSC-CMs* and cardiac biopsies uncovered robust, and conserved, alterations in cardiac DNA methylation, from which 100 sites were validated using an independent cohort. Among the CpG sites identified, hypo-methylation of the *ATG* promoter was identified as a diagnostic marker of HF, wherein cg03800765 methylation (AUC = 0.986, *P* < 0.0001) was found to out-perform circulating NT-proBNP levels in differentiating heart failure. Taken together, these findings support a novel approach of indirect epigenetic testing in human HF.

**Supplementary Information:**

The online version contains supplementary material available at 10.1007/s00395-022-00954-3.

## Introduction

Heart failure (HF) is a multifaceted clinical syndrome that is diagnosed based on clinical evidence of hemodynamic insufficiency. Patients with HF initially present with nonspecific symptoms of fatigue and exertional dyspnea, warranting a broad diagnostic workup to identify the underlying cause(s). Despite its widespread use, the poor specificity of elevated circulating BNP or NT-proBNP levels limits its use as a diagnostic tool to “ruling-out” the presence of HF [54]. Techniques to characterize the functional consequences of cardiac dysfunction, including non-invasive imaging and functional tests, provide some prognostic insights, but no molecular tests are yet available to diagnose HF. A new approach to diagnose HF and predict outcome is therefore needed, one which reflects the molecular foundations of its pathogenesis.

Although lifestyle and genetic factors have been shown to confer HF risk, their convergence onto epigenetic machinery presents an opportunity for diagnostic testing. Genome-wide association studies have uncovered thousands of causal genetic mutations [4], but the clinical value of these discoveries is limited by both the relative infrequency and pleiotropy of monogenic cardiomyopathies [25]. Environmental and behavioral factors such as obesity [1], diabetes mellitus [18, 19], and hypertension [39] are far more prevalent risk factors for HF, though the synergistic effects of environmental exposures and the plethora of mediators remain largely unknown. Recent studies have therefore begun to study the molecular basis of gene-environment or epigenetic interactions as underlying determinants of HF susceptibility and pathogenesis [42].

Unlike the direct epigenetic profiling of solid tumors, which has already shown promise in precision-based oncology [52], diagnostic access to myocardial tissue remains comparably limited. Epigenetic modifications, whether directly to DNA via CpG methylation or to ancillary structures including histone proteins, have been linked to pathogenesis of cardiovascular disease [12, 20, 26, 44, 49, 53]. Recent studies have uncovered robust differences in cardiac DNA methylation in patients with end-stage heart failure [11, 13, 28, 35], displaying both etiology-specific [36] and socioeconomically driven [37] effects on cardiac metabolic programs. Hence, DNA methylation may encode the complex environmental exposures, including circulatory milieu, which lead to cardiac dysfunction.

**Fig. 1:**
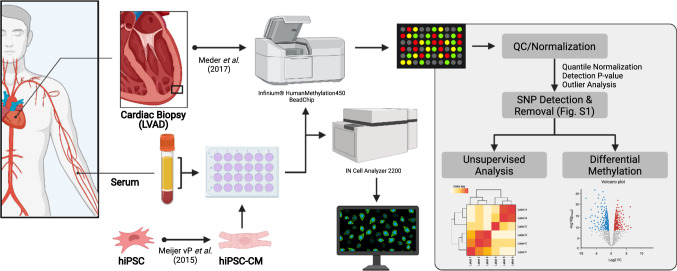
Graphical overview. Human inducible pluripotent stem cells (iPSC-CMs) were treated with plasma from either DCM (*n* = 13) or healthy (*n* = 10) subjects for 48 h. Samples were then analyzed for cell size using InCell Analyzer and submitted for methylation analysis with the Illumina^™^ Beadchip HumanMethylation450k (m450k) Array platform. Data were then cleaned and analyzed in comparison to m450k analysis of human cardiac biopsies from explanted hearts of DCM patients (*n* = 7) and non-failing donor controls (*n* = 3)

Therefore, the current study employs a novel diagnostic approach via indirect epigenetic testing to determine whether circulating factors are capable of driving epigenetic reprogramming of cardiomyocytes. The current study treated human inducible pluripotent stem cell-derived cardiomyocytes (hiPSC-CMs) with plasma collected from patients with non-ischemic HF caused by dilated cardiomyopathy (DCM, *n* = 13) and healthy donors (*n* = 10) (Fig. 1). Genome-wide analysis of array-based CpG methylation identified 49 “indirect” epigenomic markers of DCM, which were validated in a larger published cohort. Therefore, we offer preliminary evidence to support the feasibility of indirect epigenetic testing of DCM using hiPSC-CMs.

## Methods

### Ethics statement

Human studies were approved by the ethics committee and medical faculty at the Heidelberg University Hospital (Heidelberg, Germany; appl. no. S-390/2011). Informed consent was obtained for the procurement of left ventricular assist device core biopsies, and a waiver of consent was granted for tissue samples received from non-failing hearts of organ donors. Control blood samples were obtained according to the protected health information 45 C.F.R. 164.514 e2 (Bioserve) and the BCI informed consent F-641-5 (Biochain). Patient health information was acquired at time of tissue acquisition, and all human RNA-sequencing and DNA methylation array data are available upon request.

### Patient samples

All samples were obtained from and authorized by the Heidelberg University Hospital Biobank (Heidelberg, Germany). Biopsies were selected according to age and gender matching with reduced systolic left ventricular ejection fraction (LVEF) and dilatation (Supplemental Table 1). Exclusion criteria included evidence of coronary artery disease or other clinically relevant cardiac conditions. Human myocardial biopsies were obtained from patients with DCM (*n* = 7) or from non-failing donor hearts (*n* = 3), as described previously [41].

### Differentiation of human induced pluripotent stem cells into cardiomyocytes

To determine whether cardiomyocytes exhibit differences in DNA methylation in vitro, hiPSC-CMs were differentiated using an established protocol [29, 41]. Briefly, hiPSCs were harvested from Matrigel (BD Bioscience; 354,277) coated 6-well plates (Corning) and cultured with Essential 8^™^ medium (Thermo Fisher Scientific; A1517001) and ROCK inhibitor (Tocris; 1254). The hiPSCs were cultured for 3 days or until achieving a confluence of 70–90%. The medium was then replaced by RPMI1640 (Thermo Fisher Scientific; 21875-034), insulin-free B27 Supplement (Thermo Fisher Scientific; A1895601) and 10 μM CHIR99021 (Tocris; 4423) for 24 h. The next day (Day 1), the medium was changed to RPMI1640 and insulin-free B27 Supplement. 24 h later (Day 2), cells were treated with 5 μM IWP2 (Tocris, 3533) in RPMI1640 with B27 Supplement minus insulin. On Day 5, the medium was again changed to RPMI1640 plus insulin-free B27 Supplement. After Day 7 the medium was changed every two days with RPMI1640 with B27 Supplement (Thermo Fisher Scientific; 17,504,044) until day 15. To enrich cardiomyocytes, metabolic stress was induced using 4 mM lactate as described by Tohyama et al*.* [48].

Quality of isolation, and purity of *hiPSC-CMs* were assessed using cardiac troponin (cTNT) positivity versus negative control after maturation (Supplemental Fig. S1A) and after plasma treatment (Supplemental Fig. S1B). Briefly, hiPSC-CM were fixed, washed and were incubated with the primary antibody (Troponin T, Cardiac Isoform Ab-1 (Clone 13–11)) (Thermo Fischer Scientific; MS-295-P1) over night and incubated with the secondary antibody (Alexa 488 Goat anti- Ms. IgG1; Thermo Fisher Scientific A21121). Negative control is missing the first antibody (Troponin T) to show specificity of antibody binding. Quantification is performed using an automated high-throughput algorithm with InCell^®^ microscope (Supplemental Fig. S1C).

### Isolation of neonatal rat ventricular cardiomyocytes (NRVMs)

Heart pieces of 1- to 2-day-old Wistar rats were digested by a mix of collagenase (CellSystems Biotechnologie Vertriebs GmbH) and pancreatin (Sigma-Aldrich) and incubated at 37 °C for 20 min. The supernatant containing the NRVMs was sequentially collected. NRVMs were pelleted by centrifugation and re-suspended in a salt balanced solution. NRVMs were finally purified using a discontinuous Percoll gradient (GE Healthcare). Cells were re-suspended in DMEM (Sigma-Aldrich) with supplements and plated on collagen (Sigma-Aldrich) coated cell culture plates (Greiner Bio-One) [40].

### Cardiomyocyte plasma treatments

For cell size and perinuclear atrial natriuretic peptide (ANP) staining measurements, *hiPSC-CMs* were plated in octuplets on 96-well black µClear plates (Greiner Bio-One) with Matrigel (BD Bioscience) coating and NRVMs were plated on collagen. For DNA isolation, cells were plated on 12-well plates. After 24-h starvation with FCS-free medium, NRVMs and *hiPSC-CMs* were treated for 48 h with 5% patient plasma from DCM or non-failing control (CON) subjects instead, or with fetal calve serum (FCS) or FCS-free medium (“starve”).

### Cardiomyocyte immunofluorescence staining

Cardiomyocytes were fixed with paraformaldehyde (Sigma-Aldrich) after 48-h treatment. Antibodies against cardiac α-actinin (Sigma-Aldrich) and ANP (Peninsula Lab) were used sequentially overnight at 4 °C. Secondary antibodies (Thermo Fisher Scientific) were incubated for 1 h at room temperature. Nuclei were stained with DAPI (Thermo Fisher Scientific). Histological imaging and analyses were performed using an InCell Analyzer 2200 (GE Healthcare), where cell size and perinuclear ANP intensity could be measured using the automated HTS approach, which has been developed and validated by the InCell investigator software (GE Healthcare). Cell sorting results for troponin is shown in Supplemental Fig. 1A. As a proxy of stable purity after treatment of hiPSC-CMs, viable cells were quantified using the same HTS approach by counting all DAPI + cells and actinin overlay (see Supplemental Fig. 1B–C). Reproducibility of cell size measurements in different hiPSC-CM cell lines is shown in Supplemental Fig. 2A.

### HumanMethylation450k BeadChip (m450k) Array

Epigenome-wide DNA methylation studies were performed using the Illumina^®^ Beadchip HumanMethylation450k (m450k) array platform, as previously described [36]. For each assay, 500 ng DNA was bisulfite-treated before amplification, hybridization, and imaging standard to the Illumina^®^ protocol. Briefly, frozen biopsies were disrupted using the TissueRuptor (Qiagen). DNA isolation of disrupted biopsies or pelleted NRVMs and *hiPSC-CMs* was done using the QIAamp DNA Blood and Tissue Kit (Qiagen) according to the manufacturer’s protocol. DNA integrity was monitored by gel electrophoresis. Array intensity data generated via iScan^®^ were preprocessed and normalized using quantile normalization to adjust for technical differences in Type I/II array designs [23]. Total (methylated + unmethylated) signal intensity for each probe was weighed against the background signal via negative control probes to provide a statistical (*P* value) detection threshold (Supplemental Fig. S3). Possible confounding of differential methylation via overlapping SNPs was evaluated using *MethylToSNP* (0.99.0), removing 1494 CpG probes from the analysis of cardiac biopsy samples (Supplemental Fig. S4); no SNPs were detected among iPSC-CMs.

### RNA-sequencing

RNA sequencing analysis was performed as previously outlined [36], with detailed methods available as an online supplement. Briefly, RNA was isolated from iPSC-CMs using Qiazole™ reagent (Qiagen Inc., Hilden, Germany) and validated via fragment analysis (Agilent) to ensure RNA quality. Sample B2 was removed (RIN = 2.5) and was identified owing to RNA Integrity Numbers (RINs) which were 9.2 ± 1.5, with all samples achieving RINs > 7 (Supplemental Table 2). Samples were then submitted for paired-end 100 bp RNA sequencing which was performed at BGI Tech Solutions (Hong Kong, CN), where high-throughput next-generation RNA-sequencing was performed using the DNBSEQ^™^ G400 platform. Prior to alignment, adapters and low-quality (PHRED < 20, or 1% sequencing error rate) sequences were trimmed from reads files using trimgalore (0.5.0).

### Bioinformatics

All coding scripts used in the current study are available as an online supplement via GitHub data repository: https://github.com/mepepin/Indirect.Epigenomics. Differential methylation analysis was performed as previously described [36]. Differential methylation analysis was completed by fitting probe-wise linear models to the normalized log-ratios, followed by an empirical Bayesian shrinkage of probe-wise sample variance via *Minfi* (1.40.0) within the R (4.1.2) statistical computing environment [43].

For RNA-sequencing analysis, alignment of reads to the hg19 genome was accomplished using STAR (v2.7.9a), yielding ~ 95% uniquely mapped reads for all samples. Raw counts were generated using *Samtools* [21], with differential gene expression performed using *DESeq2* [22] (1.34.0) within the R (4.1.2) computing environment [38]. Dispersion estimates were determined via maximum-likelihood, which were shrunken according to an empirical Bayes approach to provide normalized count data for genes proportional to both the dispersion and sample size. Differential expression was then determined from normalized read counts via Log_2_(fold-change) using the Wald test followed by Bonferroni-adjusted *P *value for each aligned and annotated gene. From this, 2077 genes were found to be differentially expressed at *P* < 0.05.

### Statistical analysis

For all pairwise comparisons, the Shapiro–Wilk test for normality was performed to determine the most appropriate statistical test. Statistical comparisons were achieved using two-tailed *t* tests between DCM and CON in the cell size and ANP intensity as well as qPCR experiments. All data are reported as mean ± standard deviation unless otherwise specified.

## Results

### DCM patients’ plasma increases cardiomyocyte size and perinuclear ANP

**Fig. 2:**
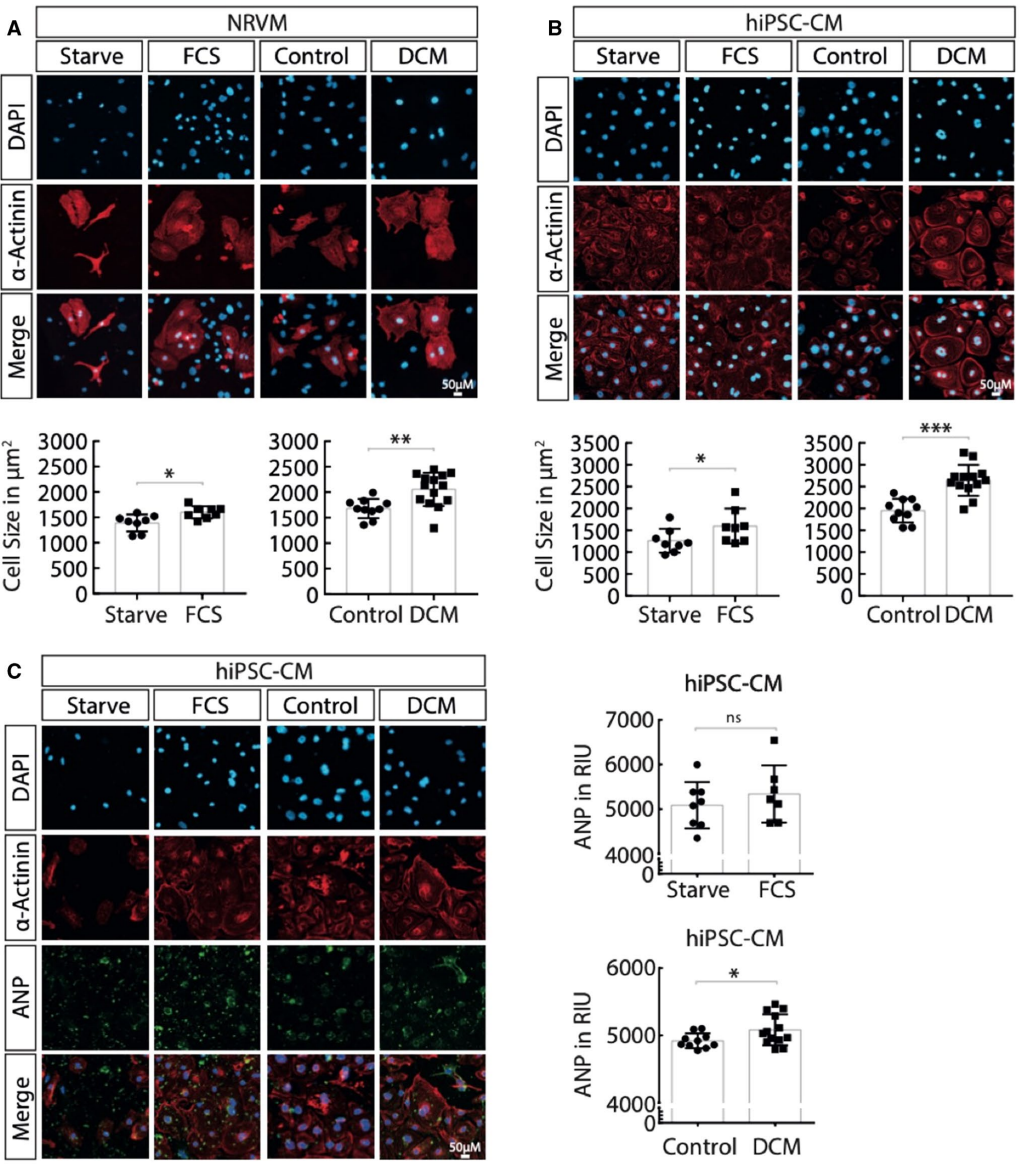
DCM patients’ plasma increases cardiomyocyte size. After 48 h of treatment with 5% plasma from dilated cardiomyopathy (DCM, *n* = 13) or healthy control (CON, *n* = 10) subjects, cell size was measured for **A** NRVMs and **B** hiPSC-CMs. **C** Representative immunocytochemistry-based quantification of atrial natriuretic peptide (ANP) performed in DCM plasma-treated (DCM) relative to control plasma-treated hiPSC-CMs co-stained for α-Actinin and DAPI (*n* = 4). Starvation vs. FCS is represented as a mean value of each well count with each approximately 1300 cells counted per well. In contrast, CTR vs. DCM is represented as a mean value of octuplets with each well counting approximately 1300 cells, hence a mean of a mean of 8 wells (a mean of 8 means, derived from approx. 1300 cells each). Student’s t-test reporting mean ± S.E.M. (*P < 0.05, **P < 0.01, ***P < 0.001)

To determine whether 48-h exposure to human plasma impacts cardiomyocyte morphology in accordance with the patients’ diagnosis of HF, cell size was quantified using the InCell^™^ automated high-content screening (HTS) assay for NRVMs (Fig. 2A) and iPSC-CMs (Fig. 2B). In both NRVMs and hiPSC-CMs, exposure to plasma from DCM patients conferred a 22% (*P* = 0.004) and 27% (*P* < 0.001) increase in cell size, respectively. Cardiomyocyte hypertrophy was reproducible, seen in repeated experiments with *hiPSC-CMs* from two additional independent cell lines (Suppl. Figure 2A). To determine whether exposure to plasma from DCM patients could reproduce pathological hallmarks of cardiac stress, an HTS approach was used to quantify both ANP abundance and its subcellular distribution within *hiPSC-CMs*. Immunohistochemical staining demonstrated greater abundance of perinuclear ANP staining in the *hiPSC-CMs* treated with DCM plasma relative to CON plasma (Fig. 2D), though neither ANP abundance nor cell size correlated with circulating NT-proBNP levels (Suppl. Figure 2B–C).

### DNA methylation changes in cardiac biopsies

**Fig. 3:**
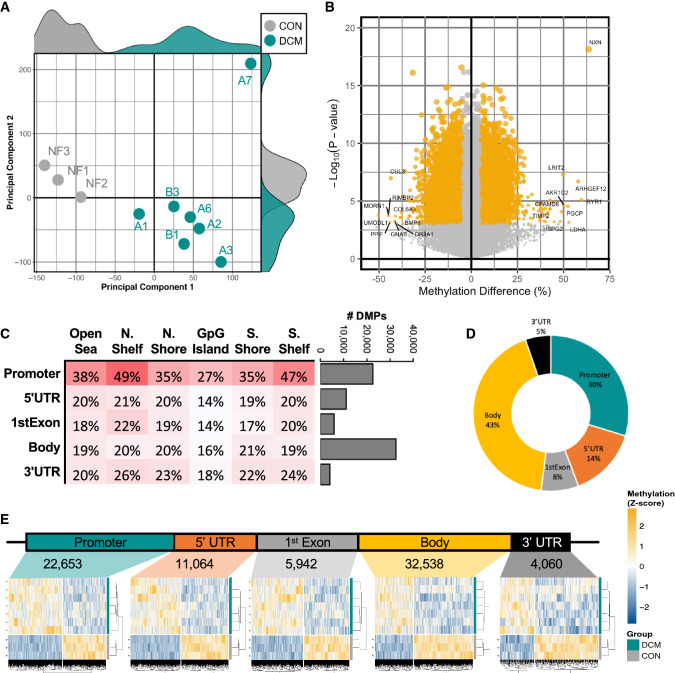
Cardiac DNA methylation in cardiac biopsies. **A** Multidimensional scaling (MDS) of top-10,000 CpG probes within the Illumina^®^ HumanMethylation450k array performed on cardiac left ventricle samples from patients with end-stage heart failure (DCM) or non-failing donor control hearts (CON). The two principal components that account from the largest variance in DNA methylation were used to generate a scatterplot, flanked by density plots of each principal component. **B** Volcano plot illustrating the robustness of CpG methylation differences, plotting (– log_10_[*P* value]) as a function of percent difference in methylation (%) in DCM vs. CON, probes exceeding *P* < 0.05 and |methylation %|> 5 highlighted in yellow. Labelled are the 10 most-robustly hyper-methylated and hypo-methylated CpG probes by % methylation. **C** Distribution of differential methylation via three-dimensional contour plot of differentially methylated CpG probes (DMPs)* categorized according to their presence within genomic (Promoter, 5’ UTR, Body, Exon–Intron boundary, or 3’ UTR) and CpG (Shelf, Shore, and Island) regions. Bar graph depicting the number of DMPs within each genomic region. **D** proportional distribution of CpG Island-associated DMPs. **E** Heatmap and hierarchical clustering of DMPs according to each genomic region. *P < 0.05

The Illumina^®^ Beadchip HumanMethylation450k array was used to quantify CpG methylation intensity of DNA isolated from biopsies of DCM (*n* = 7) and non-failing control hearts (CON, *n* = 3). Unsupervised multi-dimensional scaling (MDS) of the 10,000 most-variable CpG probes revealed a marked separation in cardiac DNA methylation signature between DCM and CON samples (Fig. 3A). Differential quantification of DCM and CON identified 84,024 differentially methylated CpG sites (DMPs) (*P* < 0.05), with the most robust alterations seen in cg02459042 (*NXN*, 63.6% hyper-methylated, *P* = 1.3 × 10^–8^) (Fig. 3B). Because DNA methylation is known to regulate gene expression in a site-dependent manner [3, 17], DMP distribution was performed according to where plotted onto both annotated gene regions (promoter, 5’UTR, gene body, and 3’UTR) as well as according to their distance from CpG Islands (CGIs) (Fig. 3C); the resulting distribution revealed that, although the greatest overall number of DMPs were located within gene bodies, a disproportionate percentage of DMPs were found within "North Shore”-associated CpG sites within the proximal promoter of adjacent genes (Fig. 3C–D). Nevertheless, strong heart failure-associated signatures of differential methylation were seen throughout the annotated genomic regions (Fig. 3E). Taken together, these findings support previously published evidence of robust epigenomic shifting in end-stage human heart failure [13, 28, 35–37].

### DNA methylation changes detected in the indirect cardiomyocyte test

**Fig. 4:**
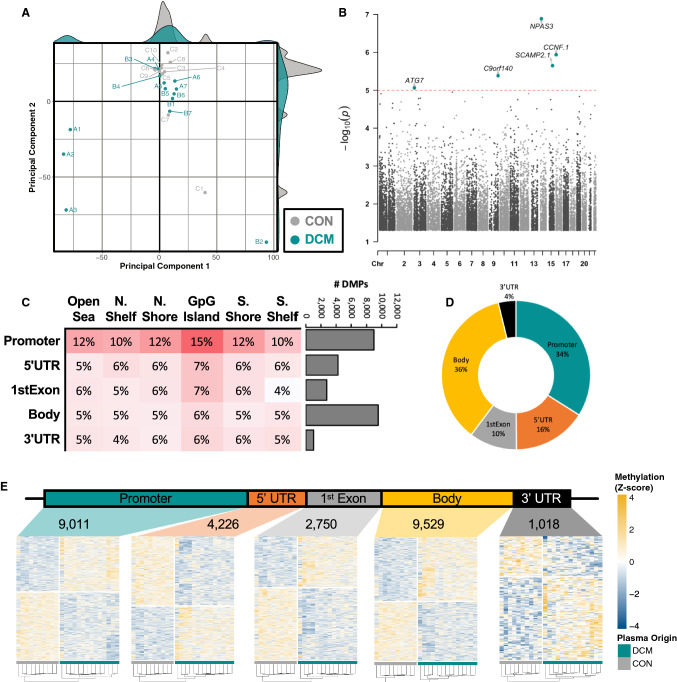
DNA methylation changes detected in the indirect cardiomyocyte test. **A** MDS plot of top-10,000 CpG probes within the Illumina^®^ HumanMethylation450k array performed on inducible pluripotent stem cell (iPSC)-derived cardiomyocytes exposed to plasma from patients with end-stage heart failure (DCM; *n* = 13) relative to plasma from healthy (CON; *n* = 10) patients. **B** Volcano plot illustrating the robustness of CpG methylation differences, plotting (- log_10_[*P* value]) as a function of percent difference in methylation (%) in DCM vs. CON, probes *P* < 0.05 and |methylation %|> 5 are highlighted in yellow. Labelled are the 10 most-robustly hyper-methylated and hypo-methylated CpG probes by % methylation. **C** Distribution of differential methylation via three-dimensional contour plot of differentially methylated CpG probes (DMPs)* categorized according to their presence within genomic (Promoter, 5’ UTR, Body, Exon–Intron boundary, or 3’ UTR) and CpG (Shelf, Shore, and Island) regions. Bar graph depicting the number of DMPs within each genomic region. **D** proportional distribution of CpG Island-associated DMPs. **E** Heatmap and hierarchical clustering of DMPs according to each genomic region. *DMPs defined via *P* < 0.05

To determine whether circulating factors are sufficient to trigger alterations in cardiac DNA methylation reminiscent of failing hearts, *hiPSC-CMs* were exposed to plasma obtained from patients with DCM or age-matched healthy control (CON) subjects. Unlike in cardiac biopsies, unsupervised clustering failed to differentiate between iPSCs exposed to DCM plasma (*n* = 13) and those with CON plasma (*n* = 10) (Fig. 4A). Nevertheless, a robust signature of differential methylation was seen between DCM and CON plasma treated *hiPSC-CMs*, with 28,381 DMPs (*P* < 0.05) detected. Of these, five DMPs achieved genome-wide significance (Fig. 4B): cg03800765 (*ATG7*, 32.4%, *P* = 8.6 × 10^–6^), cg14156314 (C9orf140, – 0.7%, *P* = 4.1 × 10^–6^), cg18502522 (*SCAMP2*, – 24.5%, 2.2 × 10^–6^), cg07561469 (*CCNF*, – 31.1%, *P* = 1.2 × 10^–6^), and cg05274755 (*NPAS3*, – 19.0%, *P* = 1.3 × 10^–7^). Furthermore, the highest proportion of DMPs relative to the m450k array were associated with promoter-associated CGIs, stressing a potential regulatory influence on adjacent coding regions (Fig. 4C). Among the CGI-associated DMPs, most were found within the promoter of adjacent coding regions (Fig. 4D), although robust differences in methylation were seen across genomic regions, as visualized via heatmap and hierarchical clustering (Fig. 4E). Taken together, these observations support that, although a global shift in DNA methylation does not distinguish between *hiPSC-CMs* treated with DCM versus CON plasma, robust alterations in DNA methylation still occur within promoter-associated CGIs.

### Common epigenetic changes detected in cardiac biopsies and by the indirect approach

**Fig. 5:**
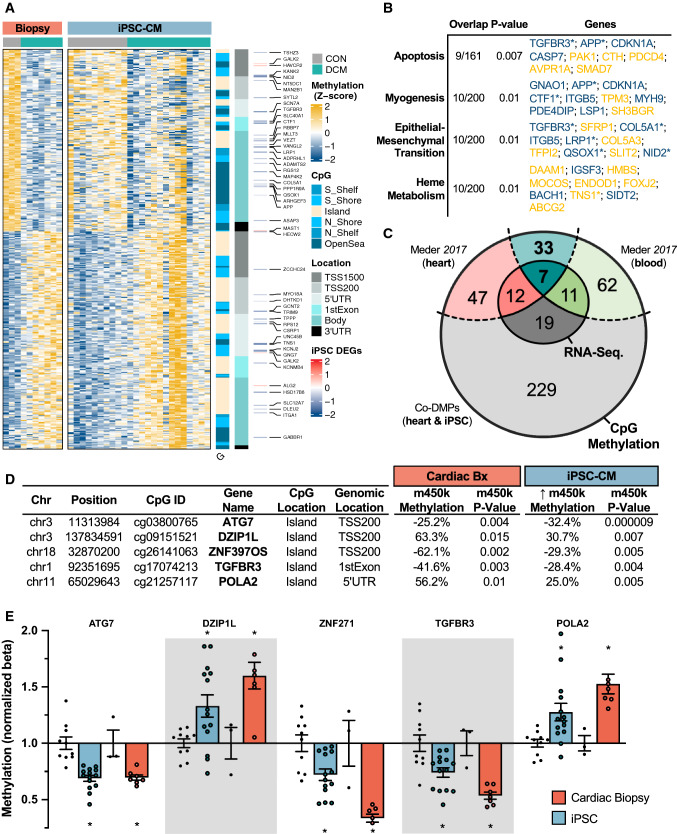
Concordant epigenetic signature of iPSC-CMs and cardiac biopsies. **A** Hierarchical clustering and heatmap visualization of 389 concordantly methylated DMPs (coDMPs)* in both cardiac tissue (red) and iPSCs (blue) treated with plasma from DCM (cyan) or healthy (grey) subjects. RNA-sequencing log_2_Fold-Change plotted alongside DNA methylation **B** Gene-set enrichment analysis of the 426 proximal genes associated with at least one of the coDMCs, using the KEGG 2020 molecular signatures database with statistical enrichment calculated using *enrichR*. **C** Venn diagram illustrating the shared DMCs between the 389 coDMPs, m450k analysis of cardiac biopsies for DCM vs. CON (*n* = 41), and m450k analysis of buffy coat for DCM vs. CON (*n* = 31). **D** Top 5 most differentially-methylated CpG sites in iPSC-CMs that could be validated using the Meder et al*.* dataset. **E** bar plot of the top 5 most robust DMCs that were present in the validation datasets. Each dot represents methylation levels of 1 well of approx. 1 million hiPSC-CMs treated with plasma, or of the available amount of myocardial tissue from patients. *P < 0.01

To identify “indirect” epigenetic loci in plasma-treated iPSC-CMs, we compared DMPs found in both myocardial and iPSC-CM analyses (Fig. 5A). Albeit a minority of co-methylated CpG sites, 389 concordant DMCs (coDMCs) associated with 426 genes were found between cardiac biopsies and iPSC-CMs. Gene set enrichment revealed disproportionate differential methylation proximal to genes associated with “Apoptosis” (*P* = 0.007, 9 DMCs), “Myogenesis” (*P* = 0.01, 10 DMCs), “Epithelial-Mesenchymal Transition” (*P* = 0.01, 10 DMCs), and “Heme Metabolism” (*P* = 0.01, 10 DMCs) pathways (Fig. 5B).

To validate DNA methylation differences observed in our cohort of human cardiac biopsies, the overlapping 389 coDMCs were compared those of a testing cohort of cardiac and blood samples from DCM (*n* = 41) and non-failing (*n* = 31) control subjects from Meder et al*.* [28] (Fig. 5C); 100 DMCs were validated in cardiac biopsies (25.7% overlap, *P* < 0.043), and 115 DMCs were also seen in blood (29.6%, *P* < 0.01). Examination of the top 5 most robustly differentially methylated CpGs in iPSC-CMs that were validated uncovered CpG island-associated CpGs located at – or near – the promoter regions for *ATG7* (cg03800765, – 32.4%, *P* = 9.0 × 10^–6^), *DZIP1L* (cg09151521, 30.7%, *P* = 0.007), *ZNF397OS* (cg26141063, – 29.3%, *P* = 0.005), *TGFBR3* (cg17074213, – 28.4%, *P* = 0.004), and *POL2A* (cg21257117, 25%, *P* = 0.005) (Fig. 5D). Plotting of each DMC revealed equivalent degrees of differential methylation at these sites between cardiac biopsies and iPSC-CMs (Fig. 5E).

To determine whether any of these CpG sites of iPSC-CMs are associated with differences in transcriptional activity, next-generation RNA-sequencing analysis was performed on the samples submitted for DNA methylation analysis. Among the 2,077 differentially expressed genes (DEGs), 49 were accompanied by proximal differential methylation (Table 1, Fig. 5C). Therefore, although the exposure of *hiPSC-CMs* to human plasma does not comprehensively recapitulate the transcriptional alterations seen in the failing myocardium, the indirect measurement of CpG methylation permits a differentiation between DCM and CON biopsies and impacts pathways known to contribute to cardiac dysfunction.

**Table 1:**
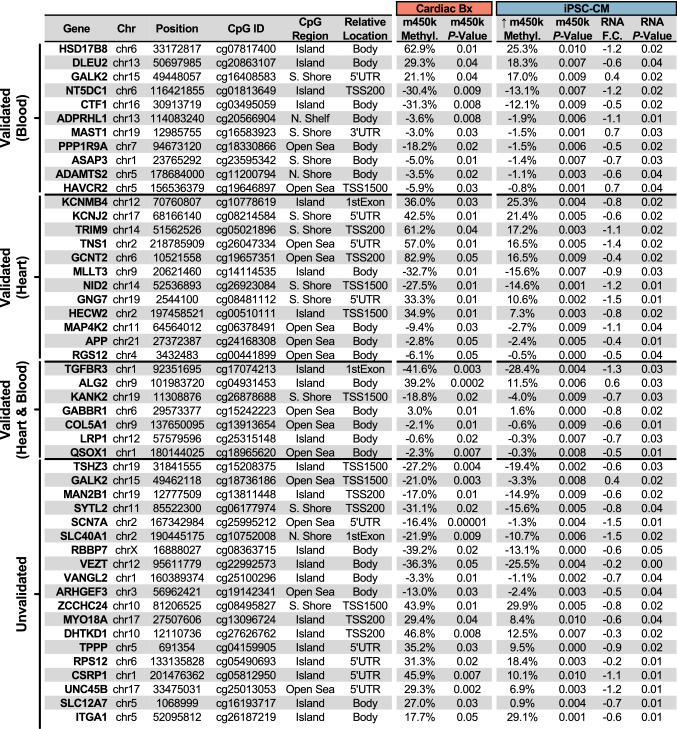
Differentially methylated genomic regions

### ATG7 as a putative epigenetic biomarker of DCM in iPSC-CMs

**Fig. 6:**
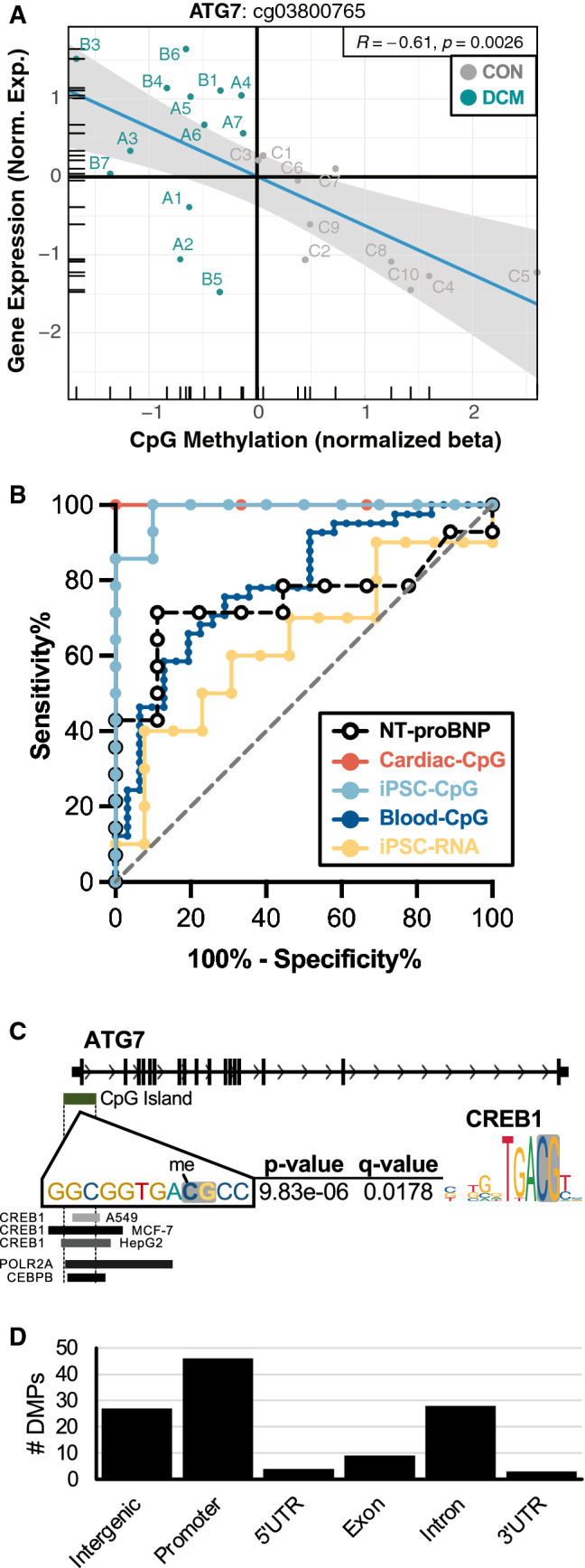
ATG7 as an indirect candidate biomarker of CREB1 activity in plasma-treated iPSCs. **A** Scatterplot correlation between CpG methylation of iPSC-CMs treated with plasma from DCM (cyan) control (grey) patients at cg03800765 and RNA-sequencing based gene expression of ATG7 (normalized counts). Also illustrated is the negative linear trend (blue line, *R* = 0.61, *P* = 0.0026) with 95% confidence region (gray). **B** Location of the CpG site cg03800765 in a CpG island adjacent to the ATG7 gene, demonstrating overlap with the CREB1 motif (MEME suite). **C** Putative downstream DMCs overlapping CREB1 response element

To better understand the transcriptional potential of single-site CpG methylation on associated gene expression, the most robustly differentially methylated CpG was taken as a use-case scenario (Fig. 6A), which displayed a strong correlation (spearman *ρ* = 0.61, *P* = 0.0026) between methylation at cg03800765 and expression of the adjacent gene *ATG7*. Area under the receiver operating characteristics (ROC) curves (AUCs) were computed for cg03800765 methylation intensity or *ATG7* expression for each dataset (Fig. 6B), revealing markedly higher AUCs for cardiac biopsy (AUC = 1.0, *P* = 0.0167) and iPSC-CM (AUC = 0.986, *P* < 0.0001) methylation relative to circulating cells (AUC = 0.789, *P* < 0.0001), iPSC-CM mRNA (AUC = 0.639, *P* = 0.264), and circulating NT-proBNP levels (AUC = 0.75, *P* = 0.05).

To identify putative upstream signaling that could be impacted by *ATG7* methylation at cg03800765, motif enrichment was performed using the MEME suite for CpG site-specific motif discovery at this DMC locus (± 10 BP). This approach identified CREB1 as a likely upstream transcriptional regulator (Fig. 6C), consistent with published evidence [32]. Downstream scanning of all DMCs for CREB1 response elements in DCM plasma-treated iPSC-CMs identified 117 overlapping DMCs; of these, 46 (39%) were located within the proximal promoter of adjacent genes (Fig. 6D). Taken together, these observations suggest that epigenetic competition of CREB1 binding may influence *ATG7* expression in DCM.

## Discussion

As a molecular readout for gene-environment interactions, epigenomic profiling offers potential for precision-based clinical diagnostics [7, 9, 24, 47, 52, 56]. For conditions in which tissue is difficult to access, including cardiovascular and neurologic diseases, clinical decision-making is forced to rely on indirect measurements, though no epigenetic biomarkers have yet been identified for diagnostic or prognostic purposes. Myocardial epigenetics has mostly been studied using biopsies from end-stage failing or post-mortem “healthy” hearts [5, 14, 31, 49, 51], thereby missing the early stages of HF in which manifestations of cardiac dysfunction may be reversible. In this study, we demonstrate the usefulness of routinely acquired blood plasma to circumvent these problems via indirect epigenetic testing of DCM patients.

### Indirect model of epigenetic testing

Although genetic heterogeneity is known to confound DNA methylation analyses, the *hiPSC-CMs* used in this study were generated from a single healthy adult of European ancestry, thereby circumventing genetic confounding. Treatment of iPSC-CMs with patient plasma induced both cellular hypertrophy and perinuclear ANP accumulation, both of which reflect properties of failing myocardium. Similarly, DNA methylation analysis identified 389 concordant DMPs (Fig. 5A), enriching pathways known to be disrupted in HF (Fig. 5B); among these, 100 DMPs (25.7%) were validated in a larger independent cohort of DCM (*n* = 41) [28]. Although we identify many promising candidates (Table 1), cg03800765 methylation exhibited superior diagnostic performance to both circulating NT-proBNP levels and *ATG7* expression in our cohort (Fig. 6B). Therefore, although future studies are needed to establish its clinical usefulness, we provide the conceptual basis for indirect epigenetic testing in HF.

### Circulating factors in heart failure

Despite the robust phenotypic and epigenetic consequences that were observed following plasma treatments, it remains unknown which circulating factor(s) is/are ultimately responsible. Their identification could enable direct measurement of plasma; however, we hypothesize that cardiomyocyte phenotype is dictated by a circulatory milieu that converges onto epigenetic machinery. Cytokines have been found to predict cardiac functional improvement on mechanical circulatory support [8]. MicroRNAs have been implicated as mediators of circulating cardiovascular risk [10]. Cardiac exosomes have also emerged as possible molecular vehicles that facilitate crosstalk between the heart and end-organ tissues [16]. A recent study by Mentowski et al*.* demonstrated that engineered exosomes can stimulate cardiomyocyte hypertrophy [30]. Therefore, the indirect testing of cardiomyocyte epigenetics may permit a collective assessment of these factors and potentially influence myocardial disease fate. Therefore, we hypothesize that the measurement of epigenetic consequences may be superior in predicting cardiovascular disease.

### DNA methylation as a proxy of HF diagnosis and outcome

Our analysis uncovered robust differential methylation cg03800765 in both iPSC-CMs (– 32.4%, *P* = 9.0 × 10^–6^) and cardiac biopsies (– 25.2%, *P* = 0.004), a CpG site located within a promoter-associated CpG island upstream of *ATG7*. Although methylation at this site was negatively correlated with *ATG7* expression (*P* = 0.0026), only cg03800765 methylation was significantly predictive of patient diagnosis with HF in iPSC-CMs (*P* < 0.0001), cardiac biopsies (*P* = 0.0167), and circulating cells (*P* < 0.0001); by contrast, *ATG7* expression failed to provide any diagnostic benefit (*P* = 0.264). Furthermore, cg03800765 methylation in iPSC-CMs out-performed circulating NT-proBNP levels as a diagnostic marker, underscoring its potential usefulness via indirect epigenetic testing (Fig. 6B). Although larger clinical cohorts are needed to evaluate the potential of indirect epigenetics to predict HF risk, cg03800765 is a promising candidate.

### Autophagy and *ATG7*

The genomic region adjacent to cg03800765 encodes the ubiquitin-like modifier-activating enzyme *ATG7*, a protein involved in phagolysosome formation and mitophagy [6]. Autophagy is essential to maintaining the regenerative potential of hematopoietic progenitor cells, and controls metabolic activity via epigenetic regulation, the dysregulation of which leads to heart failure [15, 33, 34]. Although no studies have yet explored the consequences of disrupted cardiac *ATG7* expression, familial *ATG5* mutations are associated with severe cardiac hypertrophy leading to dilated cardiomyopathy by 10 months [55]. In mice, *ATG7*^−/−^ or *ATG5*^−/−^ leads to cardiomyopathy characterized by inhibited autophagy and induced mesenchymal transition and apoptosis [45, 46, 50, 57]. Conversely, in vivo overexpression *ATG7* in mice improves autophagic capacity that ameliorates desmin-related cardiomyopathy [2]. Therefore, the differential methylation of *ATG7* may represent a phenotypically pertinent observation. However, it remains to be shown whether perturbation of the *ATG7* promoter methylation indeed causes alterations in gene expression.

### Limitations

Although the current study and analysis provide novel insights into the diagnostic potential of indirect epigenomic testing, some limitations must be considered. First, DCM etiology and medication history in our cohort could not be standardized with control subjects owing to limited supply of clinical data and tissue, respectively (see Suppl. Table 1). Although the current descriptive study uncovers an indirect epigenetic signature in iPSC-CMs following treatment with plasma of HF patients, future studies should consider early, etiology-specific signatures of DNA methylation in larger cohorts to understand its diagnostic, and possibly predictive, potential in human heart failure. Different etiologies of HF (e.g. HF with preserved ejection fraction) are possibly marked by a more systemic dysregulation of circulating metabolic factors, and thus might be even more suitable for indirect testing. Lastly, incorporation of other epigenetic marks, including histone modifications that are thought to be more signal responsive [27], may further improve the clinical precision of epigenetic testing.

## Conclusion

In the current study, we provide the first evidence that circulating factors drive indirect epigenomic alterations of iPSC-CMs and may therefore be useful for diagnostic testing. Diagnostic screening of cardiac biopsies is unfeasible, whereas development and standardization of indirect epigenomic testing using blood plasma or serum may circumvent this limitation.

### Supplementary Information

Below is the link to the electronic supplementary material.

Supplemental Figure S1.: Purity of hiPS-CMs. (A) FACS sorting for cTNT shows high purity after differentiation. (B) Cell count under control conditions and after plasma treatment shows similar results suggesting stable cell purity. 1-way ANOVA was performed. Supplementary file1 (PDF 3901 KB)

Supplemental Figure S2: (A) Cell size differences measured in 2 more human inducible pluripotent stem cell derived cardiomyocytes (hiPSC-CMs) cell lines to confirm that cellular hypertrophy is not cell-line dependent. 1-way ANOVA was performed. (B) Correlation of cell size of plasma-treated neonatal rat ventricular myocytes (NRVMs) and hiPSC-CMs with NT-proBNP of respective patients. (C) Correlation of ANP intensity with hiPSC-CM cell size. Supplementary file2 (PDF 289 KB)

Supplemental Figure S3: Mean detection P-values and beta value distribution for (A) cardiac biopsies from DCM (orange) and CON (green) subjects and (B) iPSC-CMs treated with plasma from DCM (orange) or CON (reen) subjects. Figures generated using Methylkit (1.20.0) in in R (4.0.5). Supplementary file3 (PDF 236 KB)

Supplemental Figure S4: (A) Putative SNPs. Computational identification of putative single-nucleotide variants (SNPs) was accomplished using the MethylToSNP (0.99.0) algorithm in R (4.0.5). Supplementary file4 (PDF 131 KB)

Supplementary file5 (DOCX 22 KB)

Supplemental Table 2: Fragment Analysis of RNA. Supplementary file6 (CSV 1 KB)

## Abbreviations

DCM: Dilated cardiomyopathy
DMP: Differentially methylated position
DEG: Differentially expressed gene
HF: Heart failure
hiPSC-CMs: Human-induced pluripotent stem cell derived cardiomyocytes
NRVMs: Neonatal rat ventricular myocytes
