# Supplementary figures and images for: Indirect epigenetic testing identifies a diagnostic signature of cardiomyocyte DNA methylation in heart failure
Source: Basic Res Cardiol. 2023 Mar 20;118(1):9. doi: 10.1007/s00395-022-00954-3 (PMC10027651; doi:10.1007/s00395-022-00954-3)

**A**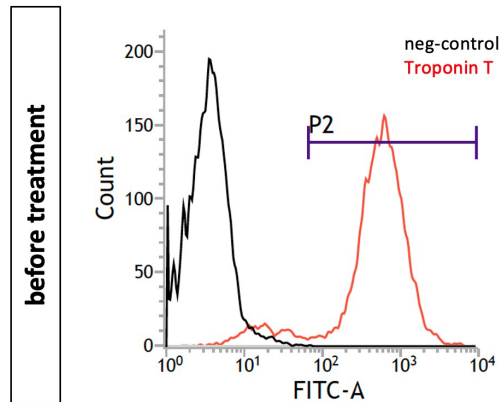**B**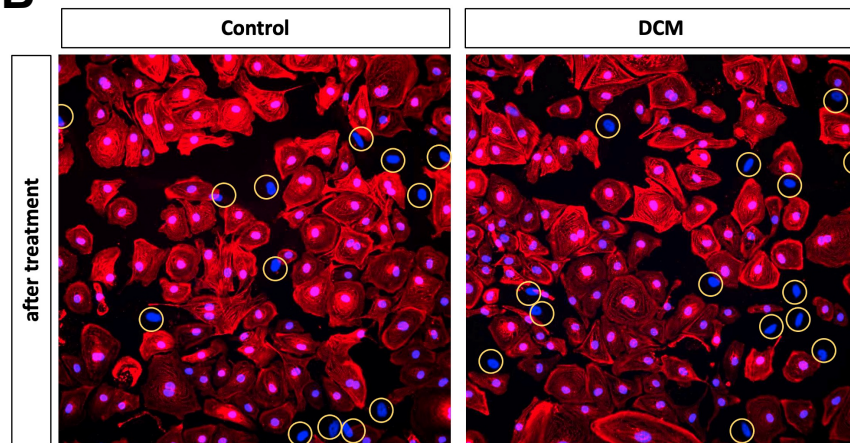**C****quantification**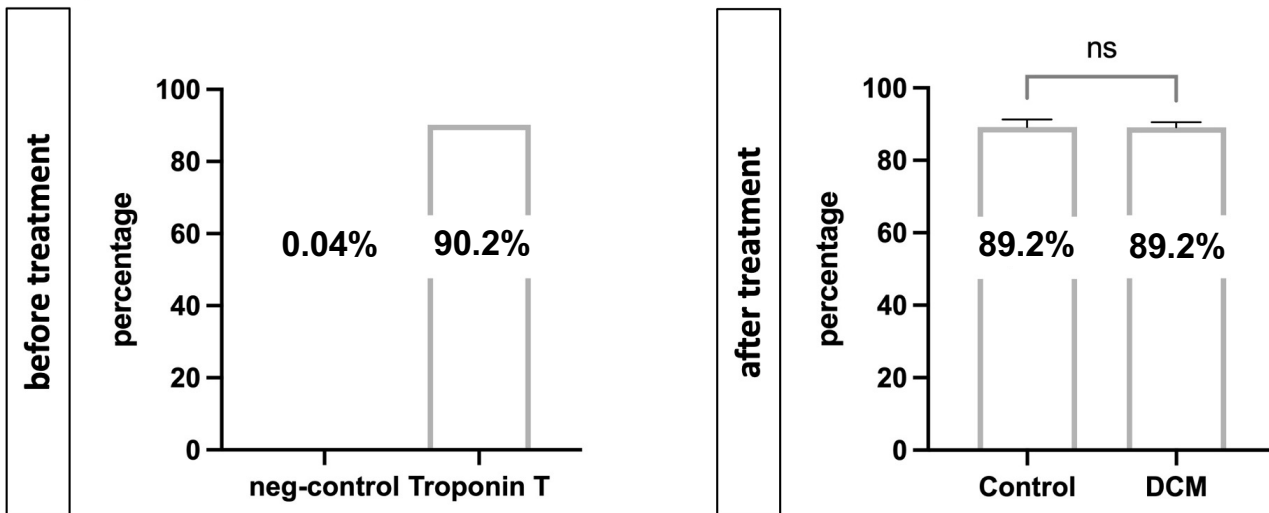

Supplement: Supplementary file 1 — Supplemental Figure S1.: Purity of hiPS-CMs. (A) FACS sorting for cTNT shows high purity after differentiation. (B) Cell count under control conditions and after plasma treatment shows similar results suggesting stable cell purity. 1-way ANOVA was performed. Supplementary file1 (PDF 3901 KB) [file 395_2022_954_MOESM1_ESM.pdf]

**A**

Line 100

Line 15

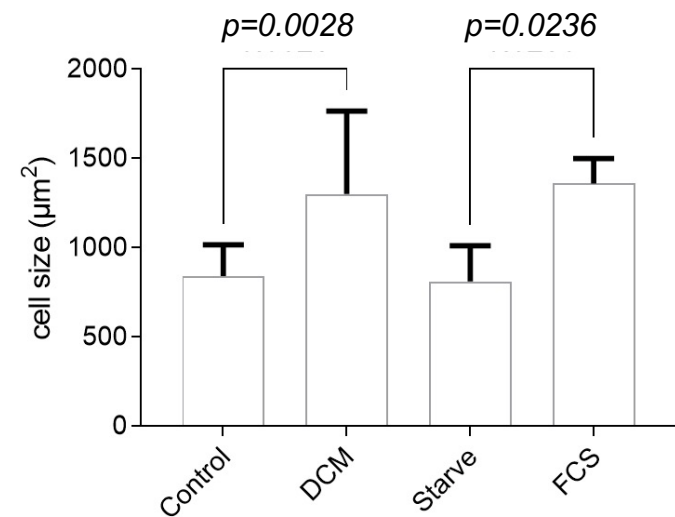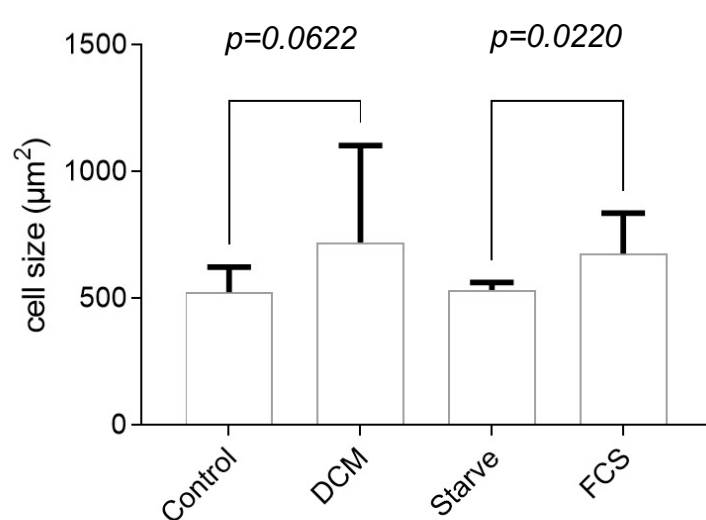**B**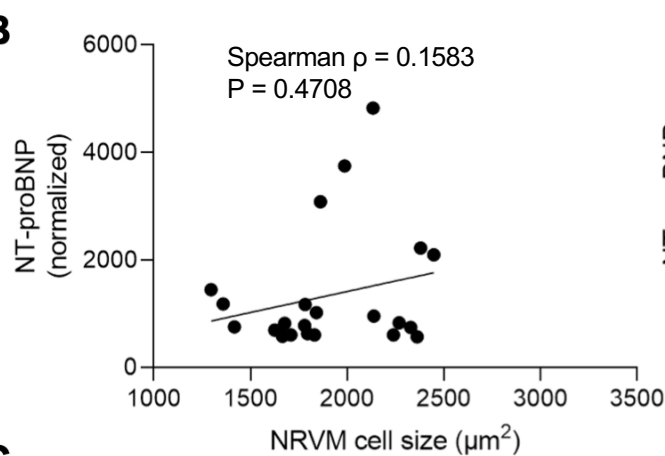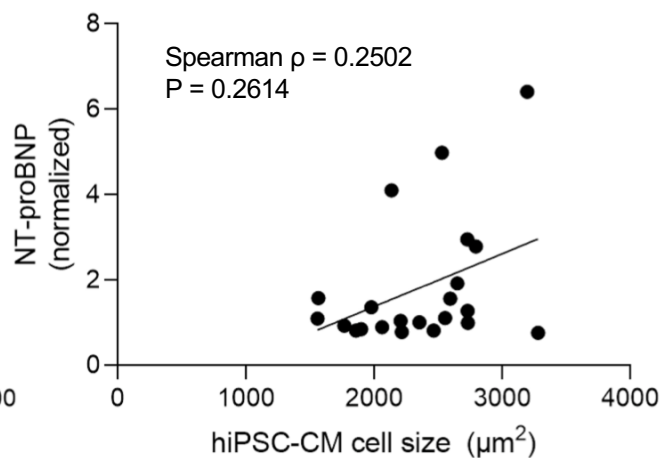**C**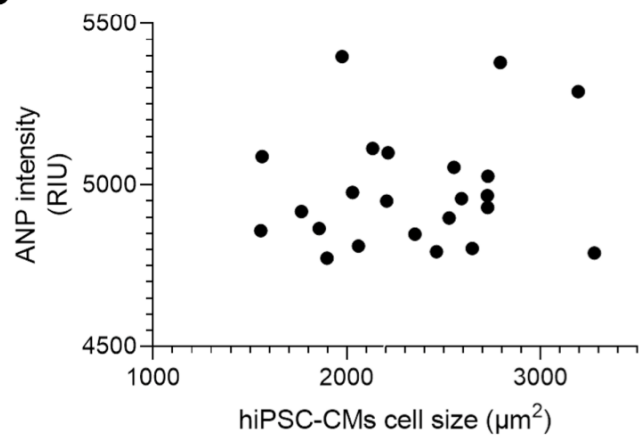

Supplement: Supplementary file 2 — Supplemental Figure S2: (A) Cell size differences measured in 2 more human inducible pluripotent stem cell derived cardiomyocytes (hiPSC-CMs) cell lines to confirm that cellular hypertrophy is not cell-line dependent. 1-way ANOVA was performed. (B) Correlation of cell size of plasma-treated neonatal rat ventricular myocytes (NRVMs) and hiPSC-CMs with NT-proBNP of respective patients. (C) Correlation of ANP intensity with hiPSC-CM cell size. Supplementary file2 (PDF 289 KB) [file 395_2022_954_MOESM2_ESM.pdf]

**A**

### Putative SNPs (477 CpGs)

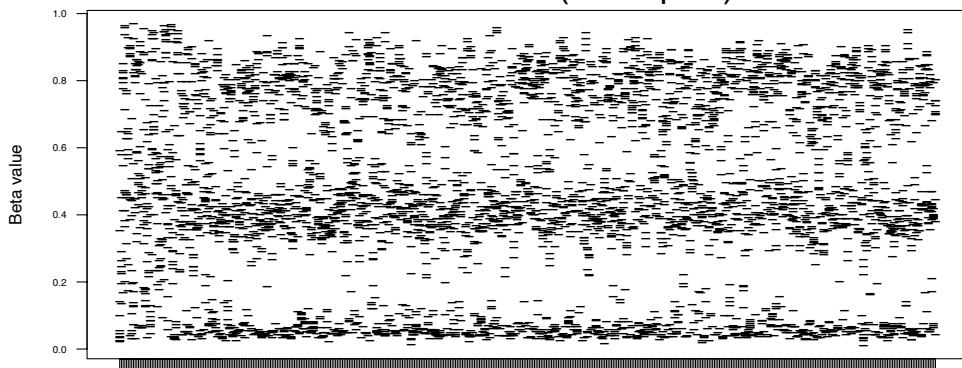**B**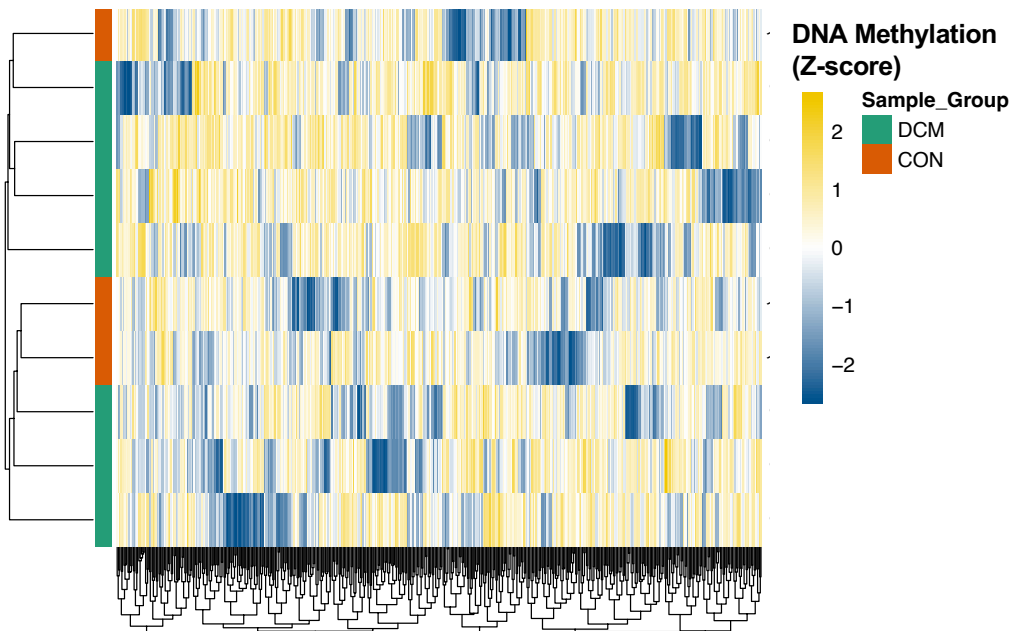

Supplement: Supplementary file 4 — Supplemental Figure S4: (A) Putative SNPs. Computational identification of putative single-nucleotide variants (SNPs) was accomplished using the MethylToSNP (0.99.0) algorithm in R (4.0.5). Supplementary file4 (PDF 131 KB) [file 395_2022_954_MOESM4_ESM.pdf]
