# Supplementary material for: Indirect epigenetic testing identifies a diagnostic signature of cardiomyocyte DNA methylation in heart failure
Source: Basic Res Cardiol. 2023 Mar 20;118(1):9. doi: 10.1007/s00395-022-00954-3 (PMC10027651; doi:10.1007/s00395-022-00954-3)
Supplement: Supplementary file 5 — Supplementary file5 (DOCX 22 KB) [file 395_2022_954_MOESM5_ESM.docx]

**Suppl. Table 1: Baseline characteristics of included patients.**

**A**

|  | ***Number (n)*** | ***Age (years)*** | ***Gender*** | ***NT-proBNP (ng/L)*** | ***Ejection Fraction (EF)*** | ***NYHA class*** |
| --- | --- | --- | --- | --- | --- | --- |
| **DCM** | 14 | 53.6 ±14.2 | 12:2 (M:F) | 31854.3 ±72929.1 | 10-50% | II-III |
| **control** | 10 | 55.3 ±15.9 | 6:4 (M:F) | 112.5±70.7 | ≥55% | not applicable |

**B**

| **DCM patients (n=14)** | ***Age (years)*** | ***Gender*** | ***NT-proBNP (ng/L)*** | ***Ejection Fraction (EF)**** | ***NYHA class*** | ***Comorbidities*** | ***Medications*** |
| --- | --- | --- | --- | --- | --- | --- | --- |
| **1** | 60.3 | M | 440 | °II-°III | II | Listed for high-urgency heart transplant | ramipril, metoprolol succinate, torasemide, spironolactone |
| **2** | 81.5 | M | n.a. | °II-°III | II | Multiple hospitalizations for decompensated heart failure | phenprocoumon, ramipril, digitoxin, spironolactone, torasemide, hydrochlorothiazide, metoprolol succinate |
| **3** | 58.5 | M | n.a. | °III | II | Transplant recommended because of multiple VTs, MR °II | bisoprolol, ASA, glimepiride, ramipril, pantoprazole, amiodarone |
| **4** | 37.9 | M | n.a. | °II-°III | II | Multiple hospitalizations for decompensated heart failure | n.a. |
| **5** | 44.8 | M | 2103 | °III | III | Listed for transplantation, ICD discharge due to VT | mexilitil, amiodarone, carvedilol, ramipril, aliskiren, torasemide, spironolactone, doxazosin, amlodipine, magnesium, potassium, pantoprazole |
| **6** | 79.2 | M | 979 | °II-°III | II-III | CRT-D Implantation, Infection of CRT-D | n.a. |
| **7** | 53.7 | F | 208223 | °III | II-III | Transiently listed for combined kidney and heart transplant, cachexia, alcohol abuse, terminal renal failure | n.a. |
| **8 ⱡ** | 61.3 | M | 272 | °II | II | ICD implantation, Diabetes type II, Depression, Sleep Apnoe Syndrome, COPD | ASA, carvedilol, ramipril, pravastatin, insulin |
| **9 ⱡ** | 61.3 | M | n.a. | °III | II | ASD-Occlusion, TIA | n.a. |
| **10 ⱡ** | 58.6 | M | 700 | °III | III | Immunapharesis, atrial fibrillation | phenprocoumon, carvedilol, ramipril, candesartan, digitoxin, torasemide |
| **11 ⱡ** | 47.6 | M |  | °I-°II | II | MVR, atrial flutter and fibrillation, hypothyreodism | L-thyroxin, ramipril, metoprolol succinate |
| **12 ⱡ** | 60.6 | M | n.a. | °III | II | Immunapharesis, atrial fibrillation | amiodarone, metoprolol succinate, candesartan |
| **13 ⱡ** | 34.3 | F | 468 | °II | II | Postpartum or postmyocarditic DCM, ICD discharge due to VT | n.a. |
| **14 ⱡ** | 34.0 | M | 10172 | °III | II-III | Myocarditis, ICD implantation, s/p immunoabsorption | phenprocoumon, carvedilol, amiodarone, candesartan, torasemide, citalopram, topiramate, pantoprazole |

**C**

| **control patients (n=10)** | ***Age (years)*** | ***Sex*** | ***NT-proBNP (ng/L)*** | ***Ejection Fraction (EF)**** | ***NYHA class*** | ***Comorbidities*** | ***Medications*** |
| --- | --- | --- | --- | --- | --- | --- | --- |
| **1** | 45.1 | M | n.a. | ≥55% | I | noCAD | n.a. |
| **2** | 63.8 | M | n.a. | ≥55% | I | artHTN, DM Typ 2, noCAD | ASA, ramipril, spironolactone, simvastatin, opipramol |
| **3** | 50.3 | F | n.a. | ≥55% | I | Nephrolithiasis, CAD excluded | ASA |
| **4** | 48.9 | F | 168 | ≥55% | I | artHTN, CAD excluded | candesartan, hydrochlorothiazide,  amlodipine, ramipril, carvedilol,  doxazosin |
| **5** | 54.3 | M | 47 | ≥55% | I | noCAD, sleep apnoe | ASA, ramipril, allopurinol, pravastatin,  beclometasondipropionat (inhal.) |
| **6** | 83.1 | M | n.a. | ≥55% | I | noCAD, PAH | spironolactone, phenprocoumon |
| **7** | 59.3 | M | n.a. | ≥55% | I | noCAD, COPD, artHTN | ASA, bisoprolol,  tiotropium bromide (inhal.), budesonide + formoterol fumarate dihydrate (inhal.), pantoprazole |
| **8** | 76.9 | F | 179 | ≥55% | I | noCAD | ASA, bisoprolol, ramipril, pravastatin |
| **9** | 37.2 | F | 56 | ≥55% | I | artHTN | telmisartan |
| **10** | 34.4 | F | n.a. | ≥55% | I | none | none |

**Suppl. Table 1: Baseline characteristics of included patients.** (A) Comparison of DCM (n=14) and control patients (n=10) characteristics regarding age, sex ratio, N-terminal proBNP (NT-proBNP), ejection fraction (EF), and NYHA (New York Heart Association) class. (B) Individual patient characteristics in the DCM cohort including medication as well as additional diagnosis apart from the main diagnosis DCM. (C) Individual patient characteristics in the “healthy” control cohort including medication as well as selected non-DCM diagnosis. Abbreviations: artHTN= arterial hypertension, ASA=acetylsalicylic acid, ASD=atrial septum defect, CAD=coronary artery disease, COPD=chronic obstructive pulmonary disease, CRT-D=cardiac resynchronization therapy (with defibrillator), DM=Diabetes mellitus, F=female, ICD=implantable cardioverter-defibrillator, inhal.=inhalative, M=male, MR=mitral valve regurgitation, MVR=mitral valve replacement, n.a.= not available, noCAD=non-obstructive coronary artery disease, NYHA=New York Heart Association classification of heart failure Symptoms, TIA=transient ischemic attack, VT=ventricular fibrillation.

*EF: °I=54-46%, °II=45-36%, °III≤35%. ⱡ: patients whose heart tissue was used for epigenetic direct approach.
