# Supplementary material for: Indirect epigenetic testing identifies a diagnostic signature of cardiomyocyte DNA methylation in heart failure
Source: Basic Res Cardiol. 2023 Mar 20;118(1):9. doi: 10.1007/s00395-022-00954-3 (PMC10027651; doi:10.1007/s00395-022-00954-3)
Supplement: Supplementary file 3 — Supplemental Figure S3: Mean detection P-values and beta value distribution for (A) cardiac biopsies from DCM (orange) and CON (green) subjects and (B) iPSC-CMs treated with plasma from DCM (orange) or CON (reen) subjects. Figures generated using Methylkit (1.20.0) in in R (4.0.5). Supplementary file3 (PDF 236 KB) [file 395_2022_954_MOESM3_ESM.pdf]

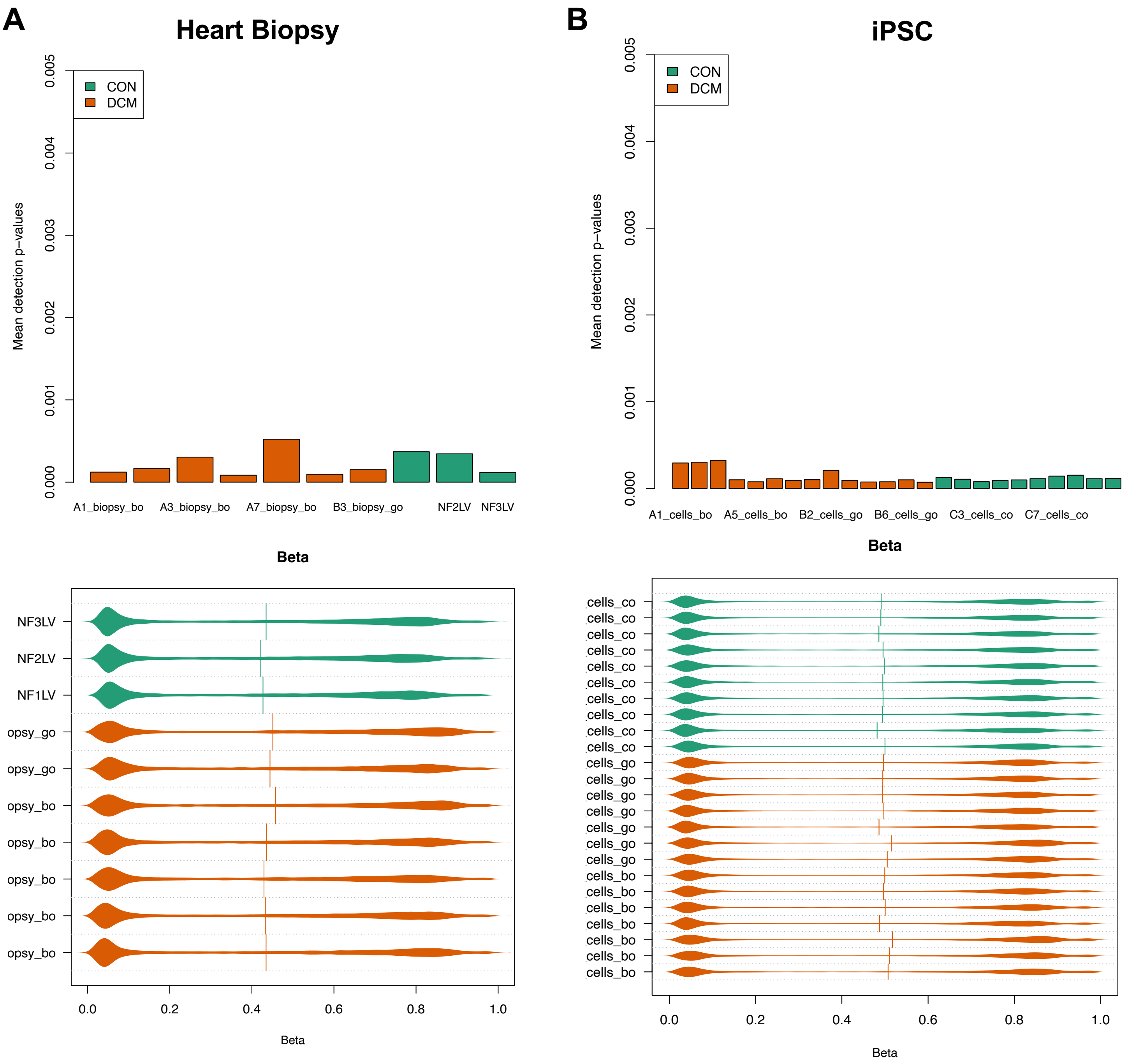

A
Heart Biopsy
CON
DCM
Mean detection p−values
0.000
0.001
0.002
0.003
0.004
0.005
A1_biopsy_bo
A3_biopsy_bo
A7_biopsy_bo
B3_biopsy_go
NF2LV
NF3LV
Beta
NF3LV
NF2LV
NF1LV
opsy_go
opsy_go
opsy_bo
opsy_bo
opsy_bo
opsy_bo
opsy_bo
0.0
0.2
0.4
0.6
0.8
1.0
Beta
B
iPSC
CON
DCM
Mean detection p−values
0.000
0.001
0.002
0.003
0.004
0.005
A1_cells_bo
A5_cells_bo
B2_cells_go
B6_cells_go
C3_cells_co
C7_cells_co
Beta
cells_co
cells_go
cells_bo
0.0
0.2
0.4
0.6
0.8
1.0
Beta
